# Marine macroalgae as food for earthworms: growth and selection experiments across ecotypes

**DOI:** 10.1007/s11356-020-07666-y

**Published:** 2020-01-10

**Authors:** Kevin Richard Butt, Camille Méline, Guénola Pérès

**Affiliations:** 1 Forensic and Applied Sciences, University of Central Lancashire, Preston, PR1 2HE UK; 2 UMR 1069 SAS (Sol Agro et Hydrosystème Spatialisation), INRA, AGROCAMPUS, 35042 Rennes, France

**Keywords:** Agroecosystems, Birch leaves, Choice chamber, Earthworm, *Fucus serratus*, Horse manure, *Laminaria digitata*, Macroalgae, Seaweed

## Abstract

Historically, subsistence farmers around the Atlantic coast of NW Europe utilized marine algae as a fertilizer in agroecosystems, a practice that continued in small areas and is now considered to have real potential for re-establishing sustainable food production systems on marginal soils. Earthworms form a significant component of soil fauna, and their ecosystem services are well-documented. Therefore, palatability of marine organic amendments to faunal detritivores of terrestrial systems is of interest. This work aimed to assess the potential for growth of *Aporrectodea caliginosa*, *Lumbricus rubellus* and *Aporrectodea longa* fed with two common macroalgae (seaweeds), *Laminaria digitata* and *Fucus serratus*. In addition, choice chambers were constructed to permit earthworm selection of these macroalgae with more conventional organic materials, horse manure (HM) and birch leaves (BL). Over a period of 2 months, earthworm species showed significantly greater mass gain with conventional food (*p* < 0.05). *Laminaria* outperformed *Fucus*, which in turn was superior to soil alone. Similarly, when given a choice, a significant preference (*p* < 0.001) was shown for the more nitrogen-rich HM and BL over the seaweeds. No removal was recorded for *A. caliginosa* when offered seaweeds only. By contrast, *L. rubellus* and *A. longa* showed significant preferences (*p* < 0.001) for *Laminaria* over *Fucus* and fresh material over degraded. These results underline an interest to profit from natural resources (seaweeds) to maintain or improve soil biological quality in marginal coastal areas.

## Introduction

The coastal Highlands of Scotland were once extensively farmed and often fertilized with seaweeds (macroalgae). This organic material was collected after winter storms or manually cut and allowed to compost. It was then incorporated into the upper parts of the ridge and furrow “lazy bed” systems (Darling 1945), the formations of which can still be observed across areas of Scotland. However, these practices largely ended with mass removal of tenant farmers during the “Highland Clearances” of the nineteenth century (see, e.g. Love 2001), when it was initially (but erroneously) thought that sheep farming would be more profitable on such land. Despite this, there are some coastal agricultural areas of Scotland and northwestern France with typically low soil fertility (e.g. the machair of South Uist) where collection and addition of macroalgae has continued (e.g. MachairLife 2013). One reason for this work was to evaluate direct effects of seaweed on soil fauna, particularly the potential as a supplement to soil organic matter and factors such as increased salts (Possinger and Amador 2016) which could have a negative effect.

Where low input or organic agroecosystems are in operation, earthworms will form a large component of the soil fauna and with microorganisms provide ecosystem services (Blouin et al. 2013) such as consumption of soil organic matter (Curry and Schmidt 2007) and soil bioturbation (Pulleman et al. 2012). Moreover, it is well-documented that the quality of food resources impacts earthworm community dynamics, structure and activity (Curry 1998; Pérès et al. 1998). An example of restarting a traditional seaweed-amended agricultural system has been undertaken in the Outer Hebrides (Knox et al. 2015), and further “lazy bed restoration” is planned. The general aim of this work was therefore to investigate the potential of macroalgae as a food for selected earthworm species with the following specific objectives:

- To determine if macroalgae might be an acceptable food for earthworms
- To compare earthworm growth rates when fed with macroalgae and more conventional food materials available in the Scottish Highlands and Islands
- To determine food preference by earthworms when macroalgae are the only resource but vary in terms of post-harvest treatment, such as washing and composting

Earthworms were selected from different ecological categories (sensu Bouché 1972), as these have different feeding strategies and in the field lead to complementary ecosystem services.

## Materials and methods

All experiments were undertaken in temperature-controlled incubators (450 l series 3, LMS Ltd., Kent, UK) in darkness at 15 °C (Lowe and Butt 2005). Initially, a growth experiment examined the use of macroalgae compared with more conventional food stocks. The same materials were then offered as food for earthworms in choice chambers.

### Food materials

The two common types of marine macroalgae used in this work, *Laminaria digitata* (Hudson) J. V. Lamouroux (commonly called kelp, tangle or oarweed) and *Fucus serratus* (L.) (saw or toothed wrack), were collected in October 2016 from the seashore at Harris on the Isle of Rum, Scotland (56.973^o^N, 6.378^o^ W). This material had been detached from its natural marine habitat by storm action. The algal biomasses were bagged and returned to the laboratory of University of Central Lancashire (UCLan), where they were frozen within 2 days of collection. For comparative purposes, more conventional food materials for earthworms, such as horse manure (oven-dried prior to use) and birch (*Betula pendula*) leaves (air-dried), were collected, as described by Butt (2011).

All food materials had percentage total C and N determined using a C,N Elemental Analyser [Carlo Erba (THERMO), FLASH EA 1112 Series] and total major elements (P, K, Ca and Mg) by inductively coupled plasma-optical emission spectrophotometry (ICP-OES) analysis after sulphuric acid digestion. Moisture content was analysed by oven drying at 105 °C for 24 h; organic matter content was determined by loss-on-ignition (550 °C for 2 h) (MAFF 1981).

### Earthworms

In addition to the algae, two species of earthworm, *Lumbricus rubellus* (Hoffmeister) (epigeic) and *Aporrectodea caliginosa* (Savigny) (endogeic), were collected from Harris on the Isle of Rum in October 2016. *L. rubellus* were directly collected from within and below piles of cattle/pony dung, whilst *A. caliginosa* were extracted with a mustard suspension from a grassland area. *Aporrectodea longa* (Ude) (anecic) were obtained by digging from Walton Hall Farm, Preston (53.746^o^N, 2.682^o^ W), in November 2016. All earthworms were then kept at 10 °C in temperature-controlled incubators at UCLan until required for experiments.

### Growth experiment

A growth experiment allowed comparison of the effect on mass change of four food treatments (two algae, horse manure, birch leaves). Plastic containers (750 ml) with lids (Lakeland, Cumbria) were used as microcosms for this experiment. Each was initially provisioned with 650 ml of moistened (20–25%) Boughton loam, a proven soil for earthworm experimentation (e.g. Butt et al. 1994). The lids were provided with mounted needle-sized air holes and containers left overnight at 15 °C to equilibrate the system. After defrosting, algal biomasses were washed 3 times in freshwater to remove surface debris and salt and then oven-dried at 105 °C, ground using a MAGIMIX 4150 W food processor and sieved to 2 mm (Boström and Lofts-Holmin 1986; Lowe and Butt 2002). The oven-dried horse manure and the air-dried birch leaves were similarly milled to pass a 2 mm sieve.

Due to the known feeding behaviours of the 3 selected earthworm species (Curry and Schmidt 2007; Lowe and Butt 2005), preparation for *A. caliginosa* (endogeic) involved mixing of the food materials with the soil in each container. Each replicate (*n* = 5 per treatment) had 10 g of food material added. For the growth experiments with *L. rubellus* (epigeic) and *A. longa* (anecic), the same amount of organic matter was rewetted and applied to the surface of the soil (*n* = 5 per treatment). Control containers had no organic matter added (soil only). To optimize earthworm density based on differences in species size, 2 immature specimens of *A. caliginosa*, 4 *L. rubellus* or 1 *A. longa* were placed into each container. Initially, across all treatments, these had mean individual masses of 0.34, 0.24 and 0.89 g, respectively. For each species, care was taken to ensure that the available earthworms were allocated across the treatments to provide equivalent starting points of mean mass.

Experimental containers were kept at 15 °C in darkness and sampled every 10 days over a period of 60 days. On sampling, the integrity of any surface organic material was maintained as well as possible. Sampling involved recording number of surviving earthworms, the individual mass of each in the containers and developmental condition (immature, with tubercula pubertatis or clitellate). Any quiescence of the *Aporrectodea* species (Sims and Gerard 1999) was also noted (seen as inactively coiled in a temporary resting position). Moisture was applied as a spray, if the soil surface appeared dry. After 30 days, the soil and organic material was fully replenished in all containers.

### Choice chamber experiments

Choice chamber experiments were conducted to test preferential selection:

1) Of four food treatments – two types of macroalgae, horse manure or birch leaves (experiment 1)

2) Of two types of macroalgae with three types of treatment (experiment 2)

Choice chambers were set up for both experiments following the methods described by Rajapaksha et al. (2013) and Ashwood et al. (2017). These consisted of circular aluminium trays (diameter 0.19 m; depth 0.025 m) with food material attached in modified Eppendorf tubes (diameter 0.01 m; depth 0.04 m). Provision of the experimental feed stocks was randomly allocated around the trays. Moistened (20–25%) Boughton loam was used as the medium to fill the trays. Earthworms were added, as described for each experiment. A sheet of pierced aluminium foil was held in place over the tray by an elastic band to provide an enclosed unit preventing earthworm and moisture escape. The choice chambers were maintained in temperature-controlled incubators at 15 °C (Lowe and Butt 2005) and examined every 3 or 4 days over a 28 day period. On examination, the Eppendorf tubes were detached from the trays and weighed. The soil surface was sprayed if it appeared to be dry. At termination of the experiment, earthworms were examined as described above.

### Experiment 1 (variety of food types)

This experiment used the same food materials as in the growth experiment (*Laminaria*, *Fucus*, horse manure and birch leaves). Two Eppendorf tubes were filled with each food stock, which had been oven-dried at 105 °C, ground and sieved at 2 mm to improve acceptability to earthworms (Boström and Lofts-Holmin 1986; Lowe and Butt 2002) (total *n* = 8 tubes per tray). Separate trays were utilized for each of the earthworm species, with the number of earthworms used based on biomass (Rajapaksha et al. 2013), i.e. 7 *A. caliginosa*, 10 *L. rubellus* and 2 *A. longa*, with a mean total tray biomass of 6.21 g, 4.66 g and 6.29 g, respectively (*n* = 5 trays per species). All earthworms were immature and prior to experimentation had been maintained at 15 °C in standard culture conditions (Lowe and Butt 2005) for 1 week. At termination of the experiment, the number of surviving earthworms and their biomasses was recorded.

### Experiment 2 (macroalgae treatments)

*Laminaria* and *Fucus* were used, but each was offered to the earthworms in three different forms. These were (a) as in Choice Chamber Experiment 1 and the growth experiment, i.e. oven-dried, ground and rewetted; (b) “fresh”-washed and cut with scissors into 2–3 mm sized pieces, after defrosting; and (c) “degraded” – treated as for (a) but then left wet for 3 weeks at 15 °C to allow degradation by fungi. With 2 tubes per food stock, a total of 12 were present for each tray. As in Experiment 1, *A. caliginosa*, *L. rubellus* and *A. longa* were used in separate trays, with five replicates per species. At termination of the experiment, the number of surviving earthworms and individual earthworm biomasses was recorded.

### Data analysis

For the growth experiment, mean mass of earthworms per vessel (*n* = 5) at the outset (day zero) was compared with final mean masses for each treatment using a one-way analysis of variance (ANOVA) with Tukey-Kramer pairwise comparisons, given that all assumptions were met.

For the choice chamber experiments, organic matter selection behaviour was determined by calculating the mass (%) remaining in the Eppendorf tubes over time. The remaining amount of litter was associated with earthworm preferences, highest remaining (%) for non-preferred and lowest remaining (%) for preferred. One-way ANOVA was used to test organic matter preference of each earthworm species separately. Normality and homogeneity of variance were tested before performing ANOVA, and if an assumption was violated with a valid reason, the regular analysis and statistical significances were confirmed with a Kruskal-Wallis test. A Tukey-Kramer multiple comparisons test was applied for all pairwise comparisons.

## Results

### Growth experiment

**Fig. 1:**
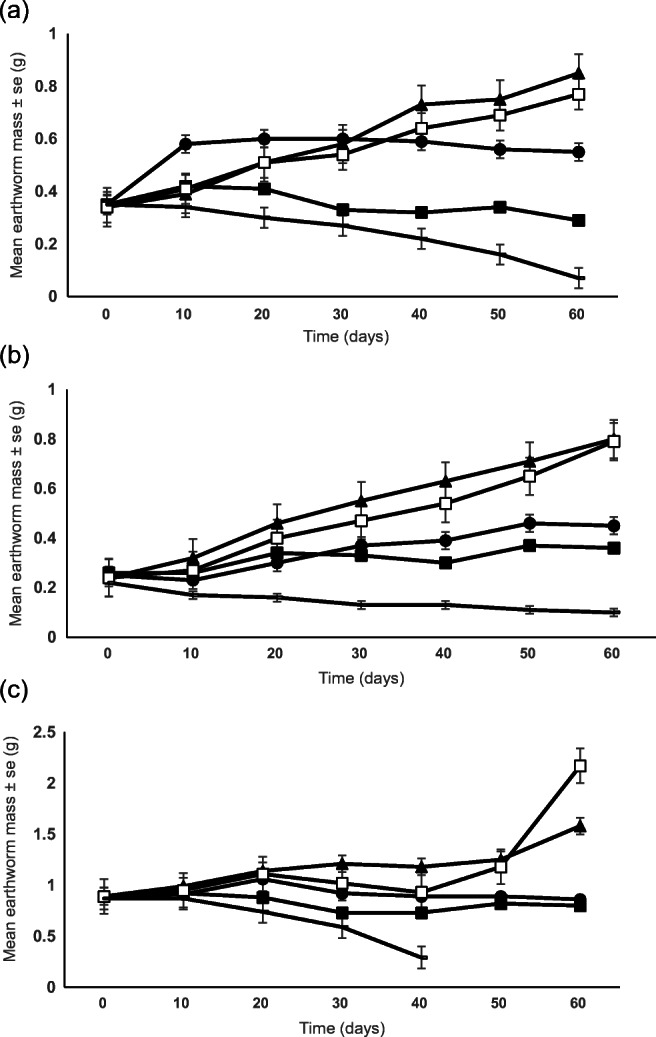
Mean (± se) mass of immature earthworms (**a**) *Aporrectodea caliginosa*, **b** *Lumbricus rubellus* and **c** *Aporrectodea longa* with different food materials (▲, horse manure; □, birch leaves; •, *Laminaria*; ▪, *Fucus*; **−**, soil only) (*n* = 5 replicates)

Significant differences in final mean masses of *A. caliginosa* were attained (*p* < 0.05) (Fig. 1a). Fed with horse manure and with birch leaves, *A. caliginosa* grew steadily, reaching mean ± SD masses of 0.85 ± 0.17 and 0.77 ± 0.11 g, respectively, after 60 days, corresponding to an increase of 150% and 126%. By comparison, growth with *Laminaria*, although positive, led to a final mean mass of 0.55 ± 0.06 g (an increase of 62%), whilst mass loss was recorded with *Fucus* (to 0.29 ± 0.04 g, a decrease of 15%) and with soil alone (to 0.07 ± 0.02 g). Most *A. caliginosa* survived, apart from when no food was supplied where mortality was 70%. In the *Fucus* food treatment, 2 earthworms were found to be quiescent at final sampling.

Growth of *L. rubellus* was positive for all food treatments, compared with the soil only control (Fig. 1b). Significantly greater growth (*p* < 0.05) was obtained with horse manure and with birch leaves (to mean masses of 0.80 ± 0.12 and 0.79 ± 0.10 g, respectively, corresponding to an increase of 233%) compared with the two algae, where a mean of 0.45 ± 0.16 g was reached for *Laminaria* and 0.36 ± 0.04 g for *Fucus* (corresponding to an increase of 87% and 50%, respectively). Overall mortality for this earthworm species was minor (8%).

Significantly greater *A. longa* growth was attained with birch leaves and horse manure than with *Laminaria* or *Fucus* (mean final masses of 2.17 ± 0.55, 1.58 ± 0.47, 0.86 ± 0.34 and 0.80 ± 0.22 g, which corresponded to an increase of 144% and 77% and a decrease of 3% and 10%, respectively) (*p* < 0.05) (Fig. 1c). Mortality of earthworms was high at 48% overall, with 100% mortality in the soil only treatment after only 40 days.

### Choice chamber experiment 1

**Fig. 2:**
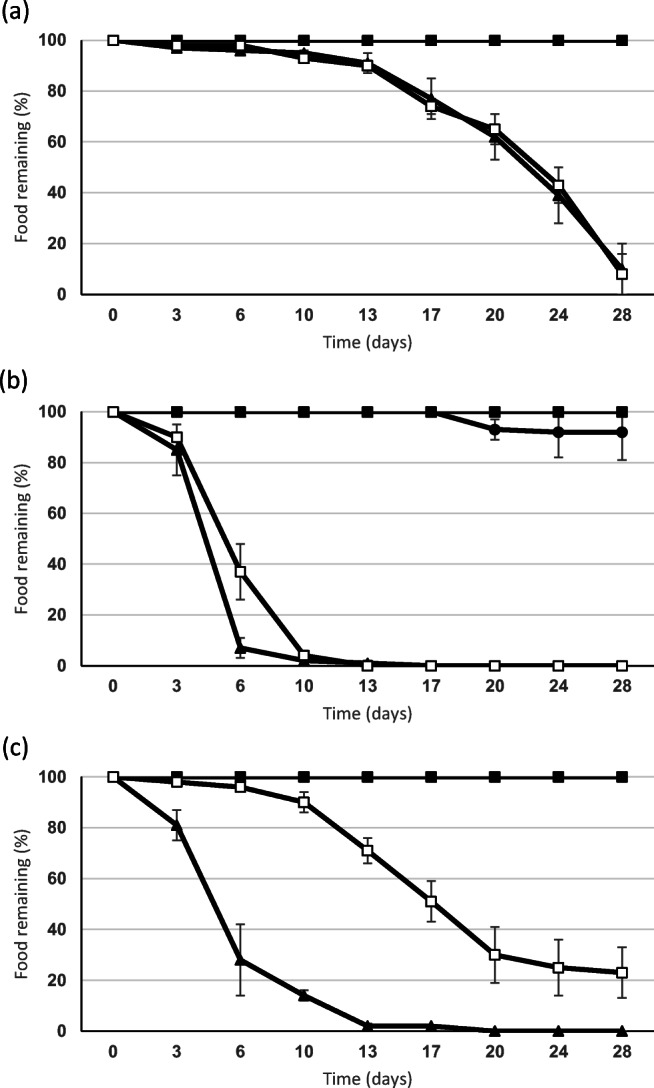
Mean (± se) choice chamber results for the earthworms (**a**) *Aporrectodea caliginosa*, **b** *Lumbricus rubellus* and **c** *Aporrectodea longa* showing amount of wet food remaining (%) over a 28 day period (▲, horse manure; □, birch leaves; •, *Laminaria*; ▪, *Fucus*) (*n* = 5 replicates)

A significant difference (*p* < 0.001) was shown for organic matter feeding by *A. caliginosa* as none of the macroalgae were removed*.* Horse manure and birch leaves were taken in very similar amounts over the experimental period, with no significant difference between these two treatments after 24 days (*p* > 0.05), with 21% and 28% remaining respectively for this species after 28 days (Fig. 2a). All *A. caliginosa* survived, decreased in mean mass from 0.88 to 0.74 g, but none were recorded in quiescence.

For *L. rubellus,* an overall significant difference was present for organic matter removal (*p* < 0.01 after 6 days; Fig. 2b). Removal of both horse manure and birch leaves was rapid, but the former was removed significantly more rapidly (*p* < 0.05 at 6 days) with the Eppendorf tubes of both emptied by 13 days. Thereafter (after day 17), a small amount of *Laminaria* was taken, but 93% of the original mass remained at experimental end. By contrast, *Fucus* was not selected (100% remained in the tubes). Mean mass of *L. rubellus* decreased from 0.47 to 0.31 g with 92% survival.

For *A. longa*, a significant difference overall was noted (*p* < 0.001) for the organic materials supplied. *A. longa* removed all of the horse manure within the first 14 days of the experimental period (Fig. 2c), with the rate of birch leaf removal increasing thereafter, with only 24% remaining after 28 days. A significant difference (*p* < 0.01) between these standard foods was recorded after 6 days, with horse manure preferred. Neither *Laminaria* nor *Fucus* was removed by *A. longa* in this experiment. All *A. longa* survived but showed a mean decrease in mass from 3.14 to 2.57 g.

### Choice chamber experiment 2

**Fig. 3:**
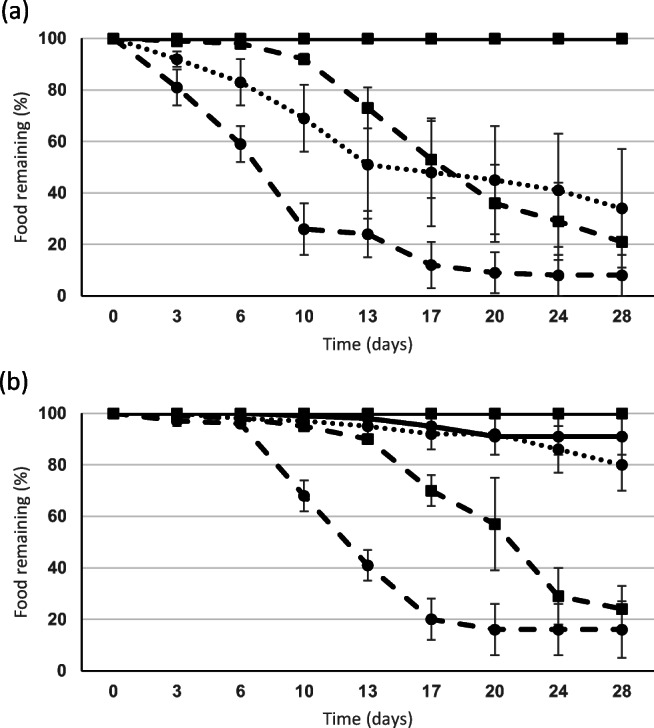
Mean (± se) choice chamber results for the earthworms (**a**) *Lumbricus rubellus* and **b** *Aporrectodea longa* showing amount of wet food remaining (%) over a 28-day period (•, *Laminaria*; ▪*, Fucus*; solid line, dried and rewetted; dashed line, fresh;, dotted line, degraded) (*n* = 5 replicates)

When offered only algae, treated in three ways, no removal activity was recorded for *A. caliginosa*. By comparison*, L. rubellus* removed large amounts of fresh *Laminaria* (more than 90%), fresh *Fucus* (80%) and degraded *Laminaria* (60%) (Fig. 3a). For this earthworm species, ANOVA after 10 days showed a significant difference for type of seaweed removed (*p* < 0.001), for treatment (*p* < 0.001) and for interaction (*p* < 0.001) between these factors. On inspection after 28 days, much of the fresh material was clearly observable within the soil of the choice chamber but had been removed from the Eppendorf tubes. By contrast, dried-ground-rewetted materials (*Laminaria* and *Fucus*), in addition to degraded *Fucus,* were not selected by *L. rubellus* (overlying horizontal lines in Fig. 3a – all remained at 100%).

*A. longa* also showed a preference for removal of fresh algae from the Eppendorf tubes (Fig. 3b). After 13 days, ANOVA showed significant differences (*p* < 0.001) for algal type, treatment and interaction. Towards the end of the experiment, small amounts of degraded (20%) and dried/rewetted (8%) *Laminaria* were removed.

All *A. longa* were active, survived and increased from mean mass of 2.53 to 2.71 g*. L. rubellus* changed little in mass during the experiment (0.30 to 0.31 g) with 70% survival, whereas *A. caliginosa* decreased from 0.72 to 0.57 g on average, with 97% survival, although 77% of this species were quiescent at experimental end.

### Analytical results

Data obtained from analysis of the food materials used in all experiments is presented in Table 1. Major variability within the food types related to C:N and some major nutrients. The macroalgae had higher C:N than the horse manure and birch leaves and much higher levels of some elements, e.g. sodium.

**Table 1:** Selected characteristics of organic materials used in feeding experiments with earthworms (unless shown, measurements are in ppm)

Food material				
	*Laminaria*	*Fucus*	Horse	Birch
			Manure	Leaves
Characteristic				
C (%)	37.45	46.46	46.76	71.42
N (%)	2.16	3.29	7.34	11.37
C:N	17:1	14:1	6:1	6:1
Na	94.84	121.91	14.04	3.09
Mg	789.97	816.53	513.33	192.29
K	191.85	224.56	69.69	76.17
Ca	7049.53	4437.7	6847.98	2463.15

## Discussion

Earthworms are known to ingest and utilize terrestrial (micro)algae as a part of their diet (e.g. Piearce 1978; Schmidt et al. 2016). Additionally, *Lumbricus terrestris* has been known to consume freshwater (macro)algae foraged from a garden pond (author’s unpublished data). Unsurprisingly, there appears to be no literature relating directly to the consumption of marine algae by terrestrial oligochaetes in agroecosystems. However, with some regard to soil dwelling earthworms, experimental work has been undertaken using macroalgae as a fertilizer in agroecosystems. Blackshaw (1989) applied a “calcareous seaweed product” to soils to assess the effects over 4 years on earthworm populations but found no evidence to support growth in population size or biomass. In a field trial, Possinger and Amador (2016) assessed the effects of applying a mixture of brown and red macroalgae on growth of sweet corn. As part of this trial, the authors also assessed earthworm populations but found no significant effects of treatments, but this was at very low (< 10 m^−2^) population densities across all experimental plots. Nevertheless, given that macroalgae (*Laminaria* in particular) have a long history of use as a fertilizer in coastal agricultural systems (e.g. Darling 1945; Grant 1961), it is likely that after some degradation, such material is consumed by earthworms.

From results obtained in the current work, it was apparent for the earthworm species selected that all had greater mass increases when fed with horse manure or birch leaves (standard food materials, e.g. Lowe and Butt 2005). However, the two types of macroalgae (specifically *Laminaria*) did permit increases in growth or at least maintenance of mass and in all cases exceeded that of the organic matter-free controls. Therefore, even with a relatively high salt content and C:N ratio due to a low percentage of nitrogen (Table 1), *L. rubellus* showed growth with both macroalgae as food, as did *A. caliginosa* with *Laminaria*. The potential negative effect of salt could be balanced by the positive effect of increased levels of some elements such as potassium and magnesium. Although data is limited, it seems that different earthworm ecological categories/species may have a range of tolerances to the use of marine macroalgae as a feed stock, something that could usefully be investigated further.

It should be noted that the quiescence recorded for *A. caliginosa* during the growth trials could have been a function of late autumn field collection and not related directly to the materials offered as food. However, such resting positions were not recorded when more conventional food materials were provided, more likely suggesting an adverse reaction to (some of) the algal biomass.

Choice chamber experiment 1 provided data that supported the findings from the growth trials. All earthworm species showed a preference for traditional food stocks, especially for horse manure and secondly for birch leaves. The short-term nature (28 days) of the experiment meant that for *A. caliginosa* and for *A. longa*, even after all horse manure was used, birch leaves were available, so the macroalgae were left untouched. *L. rubellus* showed that *Laminaria* was preferred to *Fucus*, immediately after other foods were exhausted. A higher content of calcium in *Laminaria* could perhaps explain this preference.

Results of the second choice chamber experiment showed that the fresh-washed materials, *Laminaria* or *Fucus*, were selected more compared to the other treatments, especially the dried and rewetted form. This point underlines the importance of seaweed management before use. Comparing earthworm species, *L. rubellus*, outperformed *A. longa* in terms of material removed from the tubes, with a strong preference for *Laminaria* over *Fucus*, and fresh more than degraded, in turn more than dried and rewetted. However, this was likely a function of the number of earthworms present and ecological category, with epigeics known to interact more directly with organic matter (Curry and Schmidt 2007). *A. caliginosa*, by comparison, showed no signs of feeding on the macroalgae and lost mass during the experiment, with 77% of animals quiescent at termination. Ecological category may also be critical here, as endogeic species such as *A. caliginosa* are generally geophagous (Curry and Schmidt 2007), so their natural feeding behaviour is of the soil and organic matter that it contains. Endogeics would not normally select soil-free organic matter, as would epigeic and anecic species.

The taking of “fresh” material by *L. rubellus*, but non-consumption, could have partially been a function of particle size (2–3 mm). Moving the material by mouth would be possible but perhaps not direct consumption. Dalby et al. (1998) showed that larger *A. longa* were capable of consuming particle up to 2.5 mm in diameter, but only to a very limited extent. Nevertheless, in the current experiment, removal and incorporation into the soil by *L. rubellus* shows a behaviour that would be useful to increase fertility in the field, if not to feed this species immediately. Further, pre-composting might make the material more directly acceptable to earthworms.

Agriculture-based research, such as Knox et al. (2015), has compared composted with fresh marine macroalgae (*Ascophyllum nodosum*) to assess effects on growth of potatoes, cabbages and oats. Although results from composted materials appeared to show an increase in crop yield, the authors neither reported statistical comparisons nor assessed earthworm numbers. Preliminary investigations by Possinger and Amador (2016) used un-composted mixed seaweeds to improve soil quality and found positive short term increases in active carbon. However, this was offset by increased salt levels in the soil, and effects on earthworms were inconclusive due to low community densities in the experimental field. Considered together, these studies suggest that field comparisons of effects on earthworms of composted and fresh macroalgae warrant further investigation.

The current work showed that marine macroalgae were an acceptable food for (some) earthworms and that these species exhibited mass gain when offered algae as the only form of organic amendment, although more conventional material may have been preferred. Future field-based work will examine (i) mixing macroalgae and other available organic resource such as horse manure, expecting a better food resource for earthworms from the combination; (ii) addition of marine algae, as a part of food production systems, to clarify the likely effects on existing, and potentially supplemented, earthworm populations; and (iii) the seasonal variability of the effect of algae on earthworms, with links to the seasonal variation of chemical composition (Schiener et al. 2015). Moreover, a more global approach, including the analysis of other biological properties, soil chemical and physical properties in addition to crop yield (Obriot et al. 2016), will test additional agronomic and environmental impacts of marine macroalgae addition.
